# Mobile air quality studies (MAQS) in inner cities: particulate matter PM10 levels related to different vehicle driving modes and integration of data into a geographical information program

**DOI:** 10.1186/1745-6673-7-20

**Published:** 2012-10-02

**Authors:** Stefanie Uibel, Cristian Scutaru, Daniel Mueller, Doris Klingelhoefer, Diana My Linh Hoang, Masaya Takemura, Axel Fischer, Michael F Spallek, Volker Unger, David Quarcoo, David A Groneberg

**Affiliations:** 1 The Institute of Occupational Medicine, Social Medicine and Environmental Medicine, Faculty and University School of Medicine, Goethe-University, Frankfurt, Germany; 2 The Institute of Occupational Medicine, Charité –Berlin, Medical School of the Free University Berlin and the Humboldt-University Berlin, Berlin, Germany; 3 Clinical Research Division of Allergy, Otto-Heubner-Centre, Charité –Berlin, Medical School of the Free University Berlin and the Humboldt-University Berlin, Berlin, Germany

## Abstract

**Background:**

Particulate matter (PM) is assumed to exert a major burden on public health. Most studies that address levels of PM use stationary measure systems. By contrast, only few studies measure PM concentrations under mobile conditions to analyze individual exposure situations.

**Methods:**

By combining spatial-temporal analysis with a novel vehicle-mounted sensor system, the present Mobile Air Quality Study (MAQS) aimed to analyse effects of different driving conditions in a convertible vehicle. PM10 was continuously monitored in a convertible car, driven with roof open, roof closed, but windows open, or windows closed.

**Results:**

PM10 values inside the car were nearly always higher with open roof than with roof and windows closed, whereas no difference was seen with open or closed windows. During the day PM10 values varied with high values before noon, and occasional high median values or standard deviation values due to individual factors. Vehicle speed in itself did not influence the mean value of PM10; however, at traffic speed (10 – 50 km/h) the standard deviation was large. No systematic difference was seen between PM10 values in stationary and mobile cars, nor was any PM10 difference observed between driving within or outside an environmental (low emission) zone.

**Conclusions:**

The present study has shown the feasibility of mobile PM analysis in vehicles. Individual exposure of the occupants varies depending on factors like time of day as well as ventilation of the car; other specific factors are clearly identifiably and may relate to specific PM10 sources. This system may be used to monitor individual exposure ranges and provide recommendations for preventive measurements. Although differences in PM10 levels were found under certain ventilation conditions, these differences are likely not of concern for the safety and health of passengers.

## Introduction

The Mobile Air Quality Study (MAQS) is focused on the traffic-related real time analysis of particulate matter (PM) in inner cities to quantify individual exposure [1]. PM is part of the global air pollution problem [2] resulting from the emission of air pollutants into the atmosphere by anthropogenic (i.e. industry or traffic) and natural (i.e. volcano eruptions) sources. Particulate matter currently is a key issue in environmental and occupational health research [3-5].

In traffic related particles, especially exhaust gases and tire abrasion represent the major problem. In contrast to the vast amount of research that has been carried out in the field of stationary PM monitoring networks, the level of knowledge about specific individual exposure situations is low. In this respect, sensoring systems are limited i.e. by low power supply capacities or the size of the systems and have to be adapted for use in mobile systems. This adaptation has been done resulting in the MAQS-platform usable for real time assessment of PM10 levels in realistic, mobile scenarios; this system also allows to integrate geographical data as well as air pollutant levels [1]. In this study we present data on the integration of geographical information: in pilot experiments we assessed in vehicle PM10 exposure in relation to open versus closed driving modes and PM10 levels in relation to vehicle speed.

## Methods

### MAQS-platform

The mobile platform for MAQS is a convertible car equipped as described in the study protocol [1]. It offers the advantage of assessing PM10 levels in both stationary and mobile modes. The PM10 quantitation module consisted of a power supply unit and an analysis unit and was placed on the passengers seat at the right side of the vehicle. Data were recorded and combined with the GPS position in order to achieve geographical information of the MAQS analysis [1]. PM10 values were recorded using a Grimm system (Portable Laser Aerosolspectrometer 1.109, Grimm Aerosol Technik, Ainring, Germany) with a 6 sec data interval. Vehicle speed was calculated using the GPS data.

### Route

The driving route started at Berlin - Heerstrasse outside of the environmental (low emission) zone driving eastward, entering the environmental (low emission) zone about 3 km later, until Berlin - Ernst Reuter-Platz, with a total distance of 10 km. After arriving at Ernst Reuter-Platz the vehicle turned and drove back to the starting point. For analysis the tour has been separated into the distance outside the environmental (low emission) zone (first 3 km, Heerstrasse to Stadtring) and inside the environmental (low emission) zone; also driving eastward and westward was analyzed separately.

### Monitoring

PM10 concentrations in the car were measured driving the convertible vehicle in three modes of ventilation, i.e. open roof (RO); roof closed with windows open (RCWO) and roof closed with windows closed (RCWC). Measurements started at 10.00 with each tour lasting for 28 minutes; each driving mode was repeated five times. On all tours the ventilation was set to “minimum”. After each tour the position of roof and windows was readjusted according to the settings given in Table 1.

**Table 1:** Driving intervals

**Interval**	**Start time**
**No**. / **mode of driving**	**time** (**hh**:**mm**)
1 roof open (RO)	10:00
2 roof closed - windows closed (RCWC)	10:28
3 closed roof- windows open (RCWO)	10:56
4 roof open (RO)	11:24
5 roof closed - windows closed (RCWC)	11:52
6 closed roof- windows open (RCWO)	12:20
7 roof open (RO)	12:48
8 roof closed - windows closed (RCWC)	13:16
9 closed roof- windows open (RCWO)	13:44
10 roof open (RO)	14:12
11 roof closed - windows closed (RCWC)	14:40
12 closed roof- windows open (RCWO)	15:08
13 roof open (RO)	15:36
14 closed roof- windows closed	16:04
15 closed roof- windows open	16:32

Meteorological factors and air pollution parameters were recorded by stationary sensors in Berlin. Relevant data were provided from the Berlin Luftguetemessnetz BLUME (station MC032 (=DEBE032) in 14193 Berlin Charlottenburg-Wilmersdorf, Forst Grunewald, Jagen 91; station MC0115 (= –DEBE067) in 10623 Berlin Charlottenburg-Wilmersdorf, Hardenbergplatz). These data were provided by the Senatsverwaltung Berlin and compared with data recorded in the vehicle.

### Data integration and analysis

Data integration and analysis was performed using purpose written software that integrated data from the vehicle (PM10) with GPS data (location, vehicle speed) using an ultramobile Samsung Q1 PC unit. For visualization, data points were localized using “Google Earth”.

### Statistics

All measurements were performed five times, with alternating ventilation modes. Data were analyzed using “Statview” (for Apple MacIntosh) and are expressed as mean +/- standard deviation. The Mann Whitney U-Test was used to analyze for significant differences.

## Results

PM10 levels were measured inside a convertible car with the three operating modes „roof open“ (RO), „roof closed, but windows open“ (RCWO) and „roof and windows closed“ (RCWC). In order to control for daytime specific effects like rush hour traffic or changing weather conditions, the modes were alternated (Tab. 1); all measurements were done successively on one day. Since wind direction and wind speed were assumed to play a major role, all tours were driven in both directions, e.a. west to east in the direction of the prevailing winds, and east to west.

**Figure 1:**
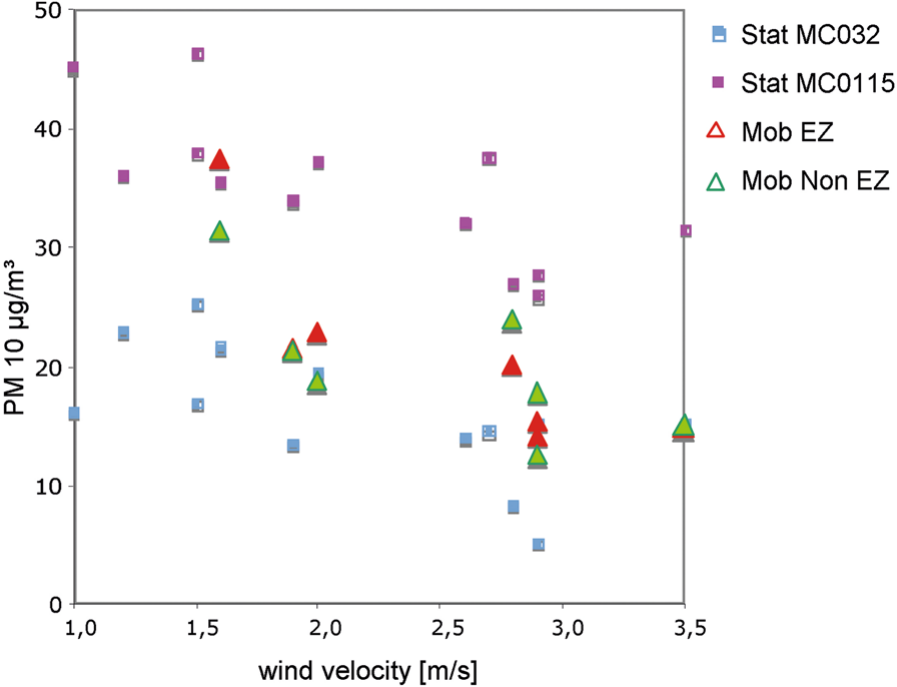
**Correlation of wind velocity and PM10 levels measured stationary and mobile.** Stat MC032 (DEBE032): Situated in 14193 Berlin, Charlottenburg-Wilmersdorf, Forst Grunewald, Jagen 91 and Stat MC0115 (DEBE067) situated in 10623 Berlin, Charlottenburg-Wilmersdorf, Hardenbergplatz). Mobile: Mob EZ, mobile analysis in the environmental zone and Mob Non EZ, mobile in the non-environmental zone

When measuring PM10 values at different wind speed, the detected values are higher at low wind speed (Figure 1). Since wind speed varied during the day, only those tours were compared and statistically analyzed that were performed sequentially; data for identical car situations, but different day times were not cumulated nor compared.

**Figure 2:**
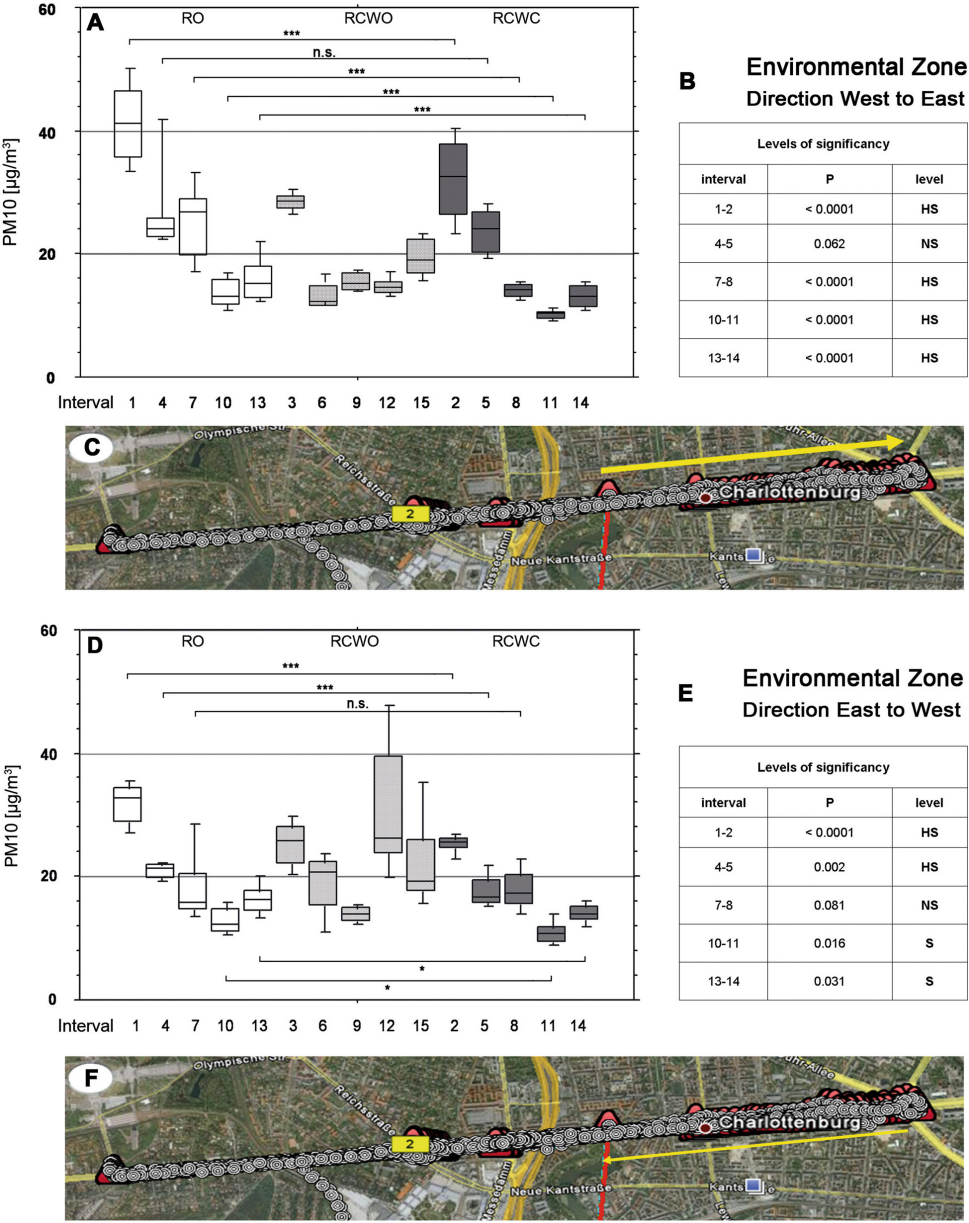
**PM 10 values within the Environmental Zone in different vehicle conditions and levels of significance.** Position of measurements within 15 intervals each of approximately 28 minutes between 10 a.m. and 5p.m.;RO = roof open, RCWO = roof closed and window open, RCWC = roof closed and window closed. **a**-**c**: direction West to East, **d**-**f**: direction East to West. Geographical picture by Google Earth

**Figure 3:**
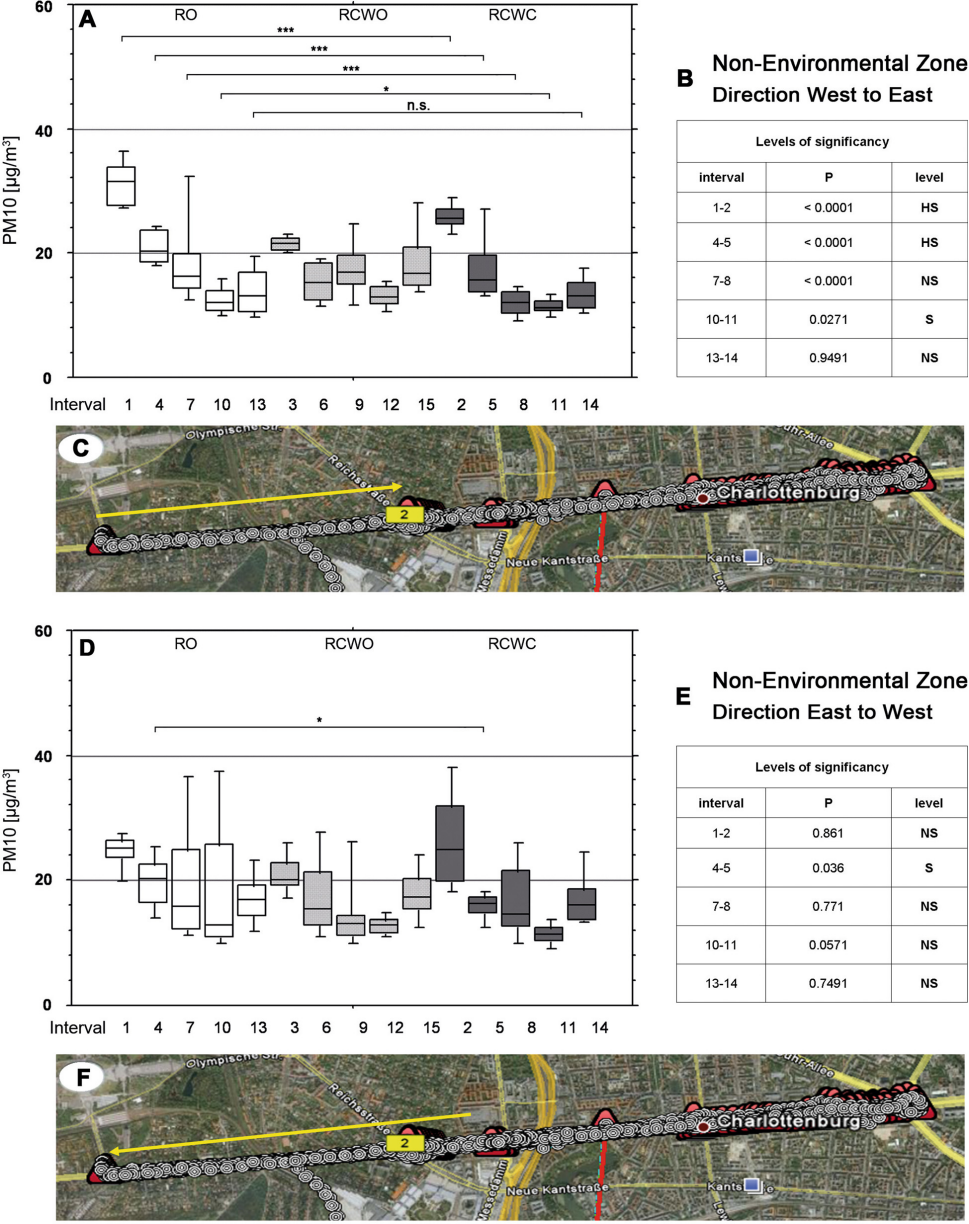
**PM 10 values within the Non**-**Environmental Zone in different vehicle conditions and levels of significance.** Position of measurements within 15 intervals each of approximately 28 minutes between 10a.m. and 5p.m.;RO = roof open, RCWO = roof closed and window open, RCWC = roof closed and window closed. **a**-**c**: direction West to East, **d**-**f**: direction Est to West. Geographical picture by Google Earth

Table 2 summarizes the mean and standard deviation of the PM10 values in the tours. Mean PM10 values are also presented in Figures 2 and 3 for the different settings (non-/environmental zones, directions, car modes). In most cases, PM10 values with open roof were significantly higher than in the mode RCWC (i.e. in the environmental zone direction west to east in four of the five tours). Similar differences were seen between RO and RCWO, whereas PM10 values were not affected when the windows were open or closed.

**Table 2:** PM10 exposure in different vehicle modes

	**Inside environmental** (**low emission**) **zone**		**Outside of environmental** (**low emission**) **zone**	
**Measurement no**. (**time of day**)	**Mean PM10 conc**., **West to East** (**μg**/**m**^**3**^)	**Mean P10 conc**., **East to West**(**μg**/**m**^**3**^)	**Mean P10 conc**., **West to East** (**μg**/**m**^**3**^)	**Mean P10 conc**., **East to West** (**μg**/**m**^**3**^)
**Roof open**				
1 (10.00 – 10.28)	41.1 ± 6.2	31.7 ± 3.1	31.2 ± 3.6	24.5 ± 2.6
4 (11.24 – 11.52)	27.2 ± 7.2	20.9 ± 1.2	20.9 ± 2.5	19.5 ± 4.1
7 (12.48 – 13.16)	25.1 ± 5.7	18.3 ± 5.9	18.6 ± 6.6	19.7 ± 9.2
10 (14.12 - 14.40)	13.6 ± 2.2	12.6 ± 2.1	12.5 ± 2.2	32.1 ± 37.9
13 (15.36 – 16.04)	15.9 ± 3.4	16.2 ± 2.4	13.8 ± 3.7	17.1 ± 3.8
**Roof closed**, **windows open**				
3 (10.56 – 11.24)	28.6 ± 1.6	25.2 ± 3.7	21.4 ± 1.1	21 ± 3.1
6 (12.20 - 12.48)	13.7 ± 2.1	18.7 ± 4.6	15.4 ± 3.0	17.2 ± 5.9
9 (13.44 – 14.12)	15.5 ± 1.4	13.8 ± 1.2	17.7 ± 4.4	17.2 ± 10.9
12 (15.08 – 15.36)	16.2 ± 6.7	41.0 ± 31.2	13.0 ± 1.7	12.6 ± 1.4
15 (16.32 – 17.00)	19.4 ± 3.0	22.6 ± 7.6	23.8 ± 18.5	17.7 ± 3.8
**Roof closed**, **windows closed**				
2 (10.28 – 10.56)	32.5 ± 6.4	25.1 ± 1.5	26.1 ± 2.6	25.9 ± 7.4
5 (11.52 – 12.20)	23.7 ± 3.6	17.6 ± 2.5	17.8 ± 5.8	15.8 ± 2.1
8 (13.16 – 13.44)	14.1 ± 1.1	17.8 ± 3.1	12.3 ± 2.5	16.4 ± 5.7
11 (14.40 – 15.08)	10.2 ± 0.7	10.9 ± 1.8	11.5 ± 1.4	11.3 ± 1.6
14 (16.04 – 16.32)	13.2 ± 1.7	13.9 ± 1.5	13.4 ± 2.6	17.7 ± 5.8

It should also be noted that PM10 values were highest during the first tours; the morning time may still have high exhaust values from the rush hour traffic. Interestingly, PM10 values were higher within the environmental (low emission) zone than outside during the first drive, independently of the roof and window mode (tour 1, 2, 3); this effect decreased at noon and was no longer observed after tour 7. It also is noteworthy that in these measurements PM10 values appeared to be comparable or lower outside the environmental (low emission) zone.

It also is obvious from the data in Table 2 that individual factors contribute largely to PM10 exposure.

Since the driving speed alters the air exchange inside a car if windows or roofs are open, PM10 values were analyzed for selected tours in relation to the speed. This analysis was restricted to tours with roof open or windows open, and the direction west to east. PM10 values and vehicle speed are compiled in Table 3.

**Table 3:** PM10 exposure at different vehicle speed

	**Inside environmental** (**low emission**) **zone**	**Outside environmental** (**low emission**) **zone**
**Measurement no**. (**time of day**)	** Mean P10 conc**., **West to East** (**μg**/**m**^**3**^)	** Mean P10 conc**., **West to East** (**μg**/**m**^**3**^)
**Roof open**		
1 (10.00 – 10.28)	A: 37.5 ± 4.1	A: 31.4 ± 2.4
	B: 34.5 ± 1.3	B: 30.3 ± 2.3
	C: 43.6 ± 6.1	C: 33.8 ± 3.4
4 (11.24 – 11.52)	A: 22.9 ± 0.6	A: 18.8 ± 1.1
	B: 23.3 ± 0.7	B: 20.4
	C: 28.9 ± 8.1	C: 22.6 ± 2.2
7 (12.48 – 13.16)	A: 20.2 ± 1.2	A: 24.0 ± 9.0
	B:-	B: 20.4 ± 0.4
	C: 25.3 ± 5.9	C: 18.8 ± 5.7
10 (14.12 - 14.40)	A: 14.1 ± 1.6	A: 12.5 ± 1.2
	B: 12.2 ± 2.7	B: 13.7 ± 2.7
	C: 13.8 ± 2.2	C: 13.4 ± 2.2
13 (15.36 – 16.04)	A: 15.5 ± 3.0	A: 17.8 ± 0.9
	B: 17.5 ± 3.8	B: 15.3 ± 5.7
	C: 16.3 ± 4.1	C: 13.5 ± 3.5
**Roof closed**, **window closed**		
2 (10.28 – 10.56)	A: 31.6 ± 6.6	A: 25.6 ± 1.0
	B: 32.5 ± 1.0	B: 26.3 ± 1.3
	C: 32.8 ± 6.0	C: 26.3 ± 3.2
5 (11.52 – 12.20)	A: 23.9 ± 3.9	A: 18.1 ± 3.6
	B: 24.8 ± 3.7	B: 14.0 ± 1.6
	C: 23.4 ± 3.6	C: 17.7 ± 6.4
8 (13.16 – 13.44)	A: 14.3 ± 0.8	A: 12.0 ± 0.4
	B:-	B:-
	C: 14.0 ± 1.2	C: 13.6 ± 2.5
11 (14.40 – 15.08)	A: 9.9 ± 0.5	A: 12.3 ± 0.9
	B: 10.5 ± 0.4	B: 12.0 ± 0.6
	C: 10.1 ± 0.8	C: 12.0 ± 1.3
14 (16.04 – 16.32)	A: 13.0 ± 2.1	A: 13.5 ± 2.6
	B: 12.8 ± 1.9	B: 16.1 ± 1.8
	C: 13.3 ± 1.7	C: 14.2 ± 2.1

Average PM10 values were calculated depending on vehicle speed.

(A) denotes mean +/- SD of PM10 at a vehicle speed of 0 – 1 km/h.

(B) at a speed between 1 and 10 km/h and (C) at a speed of 10 – 50 km/h.

**Figure 4:**
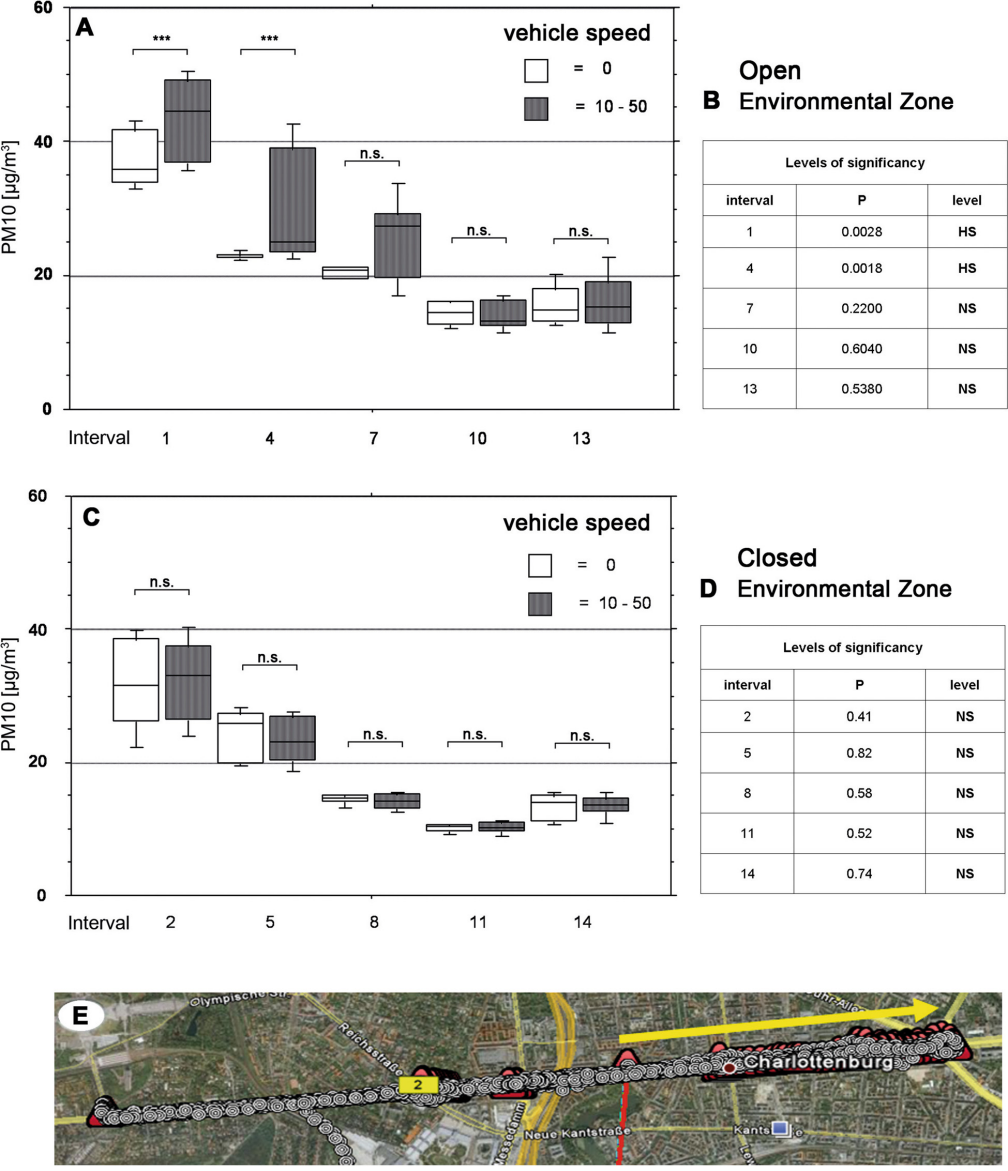
**Speed analysis in the environmental zone.** Analysis of influence of the speed on PM10 levels in the vehicle mode roof open (**A**, **B**) and vehicle mode all closed (**C**, **D**). Location of the route (**E**). Geographical picture by Google Earth

**Figure 5:**
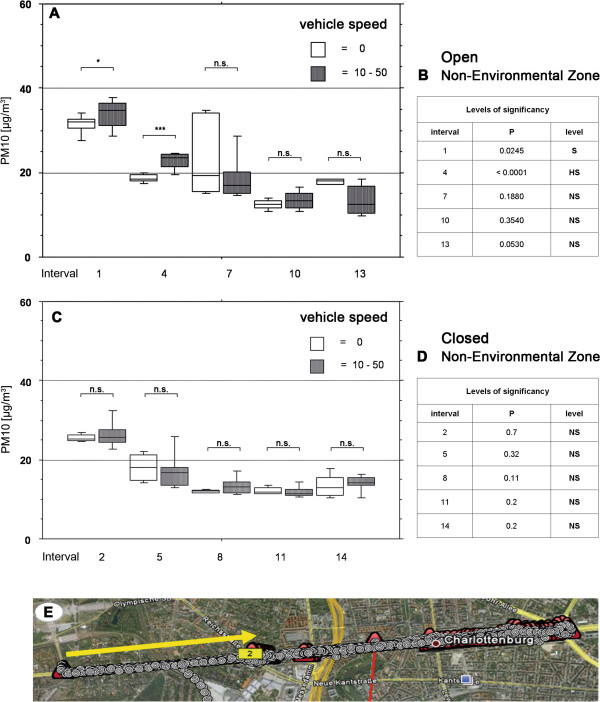
**Speed analysis in the non environmental zone.** Analysis of influence of the speed on PM10 levels in the vehicle mode roof open (**A**, **B**) and vehicle mode all closed (**C**, **D**). Location of the route (**E**). Geographical picture by Google Earth

When driving in the mode RO PM10 values in most cases appeared to be higher at higher car speed (Figures 4, 5); this effect was not observed when only the windows were open (Figures 4, 5). However, statistical analysis reached significance only in 3 out of 20 comparisons.

## Discussion

The present study used a novel approach to measure air quality using a mobile platform [1]. We assessed PM10 levels in the street situation at a real time basis in order to analyse in-vehicle-PM10 exposure with roof-open versus roof-closed driving modes.

We first addressed the influence of the wind velocity and found out that there is a correlation of velocity and PM10 values both at our mobile measurements and at the stationary BLUME analyzers. Therefore, we were not able to accumulate all data (i.e. tours 1+4+7+10+13 vs. 2+5+8+11+14) but had to compare only those measuring intervals that are adjacent, i.e. intervals 1 vs. 2 were compared, 4 vs. 5 were compared, 7 vs. 8 were compared, 10 vs. 11 were compared and 13 vs. 14 were compared, respectively. We found out that there are partly significant differences between driving cars with the roof open or the roof closed. In this respect, driving in an environmental zone in the direction West to East with the roof open produces PM10 levels between 41.1 μg/m^3^ ± 6.2 μg/m^3^ in the time interval 1. This is significantly higher than the levels observed when driving with windows and roof closed (adjacent interval 2 with 32.5 μg/m^3^ ± 6.4 μg/m^3^, p < 0.0001), indicating a barrier effect by the closed roof in this period. So far, no other studies that measured PM levels with cars presented precise data on this effect on PM10 levels have been published in the literature [1,6]. Out of 5 compared intervals all open vs. all closed, 4 differed highly significant whereas the comparison of interval 4 (all open with 27.2 μg/m^3^ ± 7.2 μg/m^3^) vs. 5 (all closed with 23.7 μg/m^3^ ± 3.6 μg/m^3^) did not differ significantly (p = 0.062). Therefore, it needs to be stated that there are still biasing factors present in our experimental set up which prevent from extrapolation of the data to other driving situations.

Since there is much public debate on the question of environmental zones and congestion charging zones over Europe [7-11], we also divided the data into non-environmental zone and environmental zone data. However, there were no large differences present and effects of environmental zones on PM10 levels can not be investigated using our MAQS approach. For this purpose, future studies should focus on data from stationary PM10 analysis prior and after the implementation of the environmental zone. Also bias factors such as weather conditions need to be taken into consideration as shown presently. In this respect, our present analysis which consisted only of data from one single day with no major weather changes apart from variations in the wind velocity between 1.5 and 3.5 m/s demonstrated that meteorological factors such as wind velocity need to be taken into account seriously.

A further aim was to investigate the effects of different vehicle speeds in closed and roof open driving modes. We compared the levels of PM10 within each driving interval. With regard to the hypothesis that a higher speed leads to a decrease in the PM10 levels measured in the vehicle, we found out that this only applies for the early intervals and only in the mode “roof open”, both in the environmental zone and the non-environmental zone. By contrast, when the roof is closed, there are no significant PM10 level differences related to the speed of the vehicle.

We can conclude that under certain circumstances, the vehicle operation mode (open/closed/speed) influences PM10 levels inside the vehicle. Concerning the biological meaning of these findings, it needs to be stated that although there are significant differences in PM10 levels, the duration of driving in open – convertible – vehicles is usually limited to summer months and non-crowded roads in a private situation. In this respect, it would be interesting to study occupational settings: i.e. the effects of opening windows on PM10 levels in trucks and lorries. So far, air quality analysis on the basis of mobile platforms did not reach large scale practical implementation [1]. Therefore, only little data is available in public databases such as the PubMed. As discussed earlier, a number of studies have used particulate matter analysis in closed vehicles [1]. In this respect, two studies assessed the exposure to fine airborne particulate matter (PM_2.5_) in closed vehicles [1,12,13]. It was reported that this may be associated with cardiovascular events and increased mortality in older and cardiac patients. Another study assessed particulate matter concentrations whilst simultaneously walking and driving 48 routes in London, UK [1,6]. Car trips were performed with closed windows and the moderate ventilation system settings. It was shown that mean exposures while walking were greatly in excess of those while driving, by a factor 4.7 for the coarse particle mass (PM10-PM2.5), 2.2 for the fine particle mass (PM2.5-PM1), 1.9 for the very fine particle mass (<PM1) and 1.4 for ultrafine particle number density [6]. It is enticing to speculate how convertible vehicle measurements would have been. With the ability of the MAQS-platform, this analysis can be performed in future. The reduced in-car exposures can be attributed to the filtration system which helped to prevent ingress of particles, so that the vehicle acted as a more-or-less independent micro-environment, insulated against much of air pollution present in the street [1,6]. In contrast to results of these studies from closed vehicles [1,12,13], exposure in open vehicles has not been investigated in great detail so far apart from the present study. In this respect, the present project may not only be used as mobile traffic pollution sensor platform but also to investigate the particulate matter exposure in open-convertible vehicles versus closed-convertible vehicles under a multitude of settings.

Concerning other mobile environmental sensing systems, a recent British project may be used as a benchmark. This project entitled Mobile Environmental Sensing System Across Grid Environments (MESSAGE) is a three-year research project that is funded jointly by the British Engineering and Physical Sciences Research Council and the British Department for Transport.

## Conclusion

The present study assessed the effects of different driving modes on in-vehicle PM10 levels. It can be summarized that the vehicle operation mode (open/closed/level of speed) can influence in-vehicle PM10 levels under distinct situations. These differences may not be of major concern for passengers’ safety and health due to the relative short duration of driving. It should be investigated on a toxicological basis whether the observed differences in the levels of PM10 may lead to effects. This may be addressed using biochemical [14-17] and molecular biology tools on the level of cell cultures and animal experiments [18,19]. Further research concerning other car types is also needed.

## Competing interests

The authors declare that they have no competing interests.

## Authors' contributions

SU, CS, DM, DK, DMLH, MT, AF, MS, VU, DQ, DG have made substantial contributions to the conception and design of the study, acquisition of the data and interpretation. They have been involved in drafting and revising the manuscript. All authors have read and approved the final manuscript.
